# Prevalence of DNA Repair Gene Mutations in Blood and Tumor Tissue and Impact on Prognosis and Treatment in HNSCC

**DOI:** 10.3390/cancers13133118

**Published:** 2021-06-22

**Authors:** Kimberly M. Burcher, Andrew T. Faucheux, Jeffrey W. Lantz, Harper L. Wilson, Arianne Abreu, Kiarash Salafian, Manisha J. Patel, Alexander H. Song, Robin M. Petro, Thomas Lycan, Cristina M. Furdui, Umit Topaloglu, Ralph B. D’Agostino, Wei Zhang, Mercedes Porosnicu

**Affiliations:** 1 Wake Forest Baptist Medical Center, Winston-Salem, NC 27157, USA; kburcher@wakehealth.edu (K.M.B.); afaucheu@wakehealth.edu (A.T.F.); jwlantz@wakehealth.edu (J.W.L.); ksalafia@wakehealth.edu (K.S.); manisha.patel10@gmail.com (M.J.P.); asong@wakehealth.edu (A.H.S.); rpetro@wakehealth.edu (R.M.P.); tlycan@wakehealth.edu (T.L.J.); cfurdui@wakehealth.edu (C.M.F.); Umit.Topaloglu@wakehealth.edu (U.T.); rdagosti@wakehealth.edu (R.B.D.J.); wezhang@wakehealth.edu (W.Z.); 2 University of Kentucky Medical Center, Lexington, KY 40536, USA; harper.wilson@uky.edu; 3 Campbell University School of Osteopathic Medicine (CUSOM), Lillington, NC 27546, USA; a_abreu0419@email.campbell.edu

**Keywords:** HNSCC, ctDNA, tDNA, DDR genes, PARP inhibitors

## Abstract

**Simple Summary:**

The DNA damage repair (DDR) gene profile is largely unexplored in head and neck squamous cell cancer (HNSCC), leaving little known about the treatment of HNSCC with PARP inhibitors. In this retrospective study, the prevalence of mutated DDR genes was studied in the tissue and/or blood samples (tDNA and ctDNA samples, respectively) of 170 patients with HNSCC. These findings were correlated with demographic and outcome data. DDR gene mutations were significantly increased in older patients, patients with primary tumors located in the larynx, patients with more advanced cancers at diagnosis and patients previously treated with chemotherapy and/or radiotherapy. Patients with primary tumors in the oropharynx were less likely to have DDR gene mutations. Patients with DDR gene mutations identified in blood samples were found to have worse survival. The combined mutational analysis in blood and tumor demonstrated a high prevalence and an important prognostic role of DDR gene mutations in HNSCC, supporting further clinical research of PARP inhibitors in the genomic guided treatment of HNSCC.

**Abstract:**

PARP inhibitors are currently approved for a limited number of cancers and targetable mutations in DNA damage repair (DDR) genes. In this single-institution retrospective study, the profiles of 170 patients with head and neck squamous cell cancer (HNSCC) and available tumor tissue DNA (tDNA) and circulating tumor DNA (ctDNA) results were analyzed for mutations in a set of 18 DDR genes as well as in gene subsets defined by technical and clinical significance. Mutations were correlated with demographic and outcome data. The addition of ctDNA to the standard tDNA analysis contributed to identification of a significantly increased incidence of patients with mutations in one or more genes in each of the study subsets of DDR genes in groups of patients older than 60 years, patients with laryngeal primaries, patients with advanced stage at diagnosis and patients previously treated with chemotherapy and/or radiotherapy. Patients with DDR gene mutations were found to be significantly less likely to have primary tumors within the in oropharynx or HPV-positive disease. Patients with ctDNA mutations in all subsets of DDR genes analyzed had significantly worse overall survival in univariate and adjusted multivariate analysis. This study underscores the utility of ctDNA analysis, alone, and in combination with tDNA, for defining the prevalence and the role of DDR gene mutations in HNSCC. Furthermore, this study fosters research promoting the utilization of PARP inhibitors in HNSCC precision oncology treatments.

## 1. Introduction

Over the past decade, next generation sequencing (NGS) of genetic material contained in blood and tissue samples (tDNA and ctDNA, respectively) has revolutionized the field of oncology [1,2,3]. Such discoveries have allowed for the treatment of non-small cell lung cancer with *EGFR, ALK* and *MET* inhibitors and basal cell cancer with hedgehog pathway inhibitors with improved outcomes. These studies have also contributed to outcome data, which have improved the management of malignant melanoma found to have mutations in *BRAF*. Many have considered the targeted treatment plans derived from the NGS of tDNA and ctDNA to be oncology’s first venture into the world of personalized medicine.

Despite the benefits of NGS in the management of many malignancies, the mutational landscape of squamous cell cancers of the head and neck (HNSCC) remains largely undescribed. This has left the field without targeted management strategies and reliable prognostication based on an individual cancer’s genetic profile [4]. Though relatively little is known, early studies regarding mutations in HNSCC have begun to lay the necessary groundwork on which clinical trials may be based. For example, data suggest that loss of function mutations in p53 [5,6,7], retinoblastoma tumor suppressor [8,9] p16 [5] and activation of p63 (all constituents of the p53 pathway) [10,11,12] are known to be frequent mutations in HNSCC, with up to 80% of patients with HNSCC experiencing loss of function mutation in p53 [6,7]. Therapies targeted to this pathway (such as adenoviral p53 gene therapy and use of small molecules to restore TP53 function/disrupt inactivation of wild-type p53) have been proposed but are yet to meet fruition [13]. Mutations in the NOTCH pathway are detected less frequently but are estimated to occur in 17% of HPV-positive and 26% of HPV-negative HNSCCs [6]. Clinical trials for patients with *NOTCH1* mutations also remain in early phases [14]. Other mutations, including those in *EGFR, MET*, RAS/RAF/MAPK and JAK/STAT pathways, have also been described in HNSCC with respective treatments in various phases of investigation [4].

A recent retrospective analysis studied 75 patients with HNSCC and revealed that 38.8% of patients had alterations in one or more DNA repair genes (limited in that study to *APC, ATM, BRCA1* and *BRCA2*). Not only was this percentage higher than previous studies would suggest, but the study was also able to demonstrate that patients with such mutations in ctDNA were associated with decreased overall survival in univariate and multivariate analysis [15]. Theoretically, cells without functional copies of these genes (and others) with a direct or an indirect role in homologous recombination repair (HRR) or the Fanconi anemia (FA) pathway are sensitive to poly (ADP-ribose) polymerase (PARP) inhibition. Genes involved in HRR resolve breaks in DNA through a PARP-independent pathway. Defects in HRR result in hypersensitivity to a number of therapeutics, including PARP inhibitors, topoisomerase inhibitors and many other DNA break inducers. The genes that encompass the FA pathway encode similarly PARP-independent DNA repair machinery utilized to resolve interstrand crosslinks. Though classically associated with hypersensitivity to platinum-based chemotherapies, defects in these genes in HNSCC have been shown to create cell lineages that rely on PARP mechanisms for DNA repair [16,17,18,19,20]. When mutations in genes involved in HRR or the FA pathway confer loss of function, PARP inhibitors can be utilized to prevent repair of breaks in DNA, ultimately leading to cell death. All clinical PARP inhibitors inhibit both PARP1 and PARP2. PARP1 repairs double-strand DNA (dsDNA) breaks and single-strand DNA (ssDNA) breaks. PARP2 repairs only ssDNA breaks. The clinical utility of PARP inhibition lies in the concept of “synthetic lethality”, in which neither a mutation in HHR genes nor PARP inhibition, alone, would be lethal to a cell, but the combination of the two factors in tumor cells ensures cell death [16].

PARP inhibitors are currently approved for breast, ovarian and pancreatic cancers carrying *BRCA1* or *BRCA2* mutations. The FDA has also approved use of PARP inhibitors for prostate cancers in which *BRCA1* or *BRCA2* or *ATM* mutations have been detected. Investigations regarding the use of PARP inhibitors in HNSCC are currently underway but are hindered by the low reported prevalence of mutations in applicable genes. Perhaps for this reason, these studies focus on their use in combination with traditional chemo- or radiotherapies rather than in cases in which NGS has directed decision making [21,22,23].

In this retrospective review, the investigators aim to validate previous findings regarding the prevalence and prognostic value of mutated DNA damage repair (DDR) genes in HNSCC utilizing combined genomic analysis performed both in blood and in tumor tissue (ctDNA and tDNA, respectively) in a larger patient population. In addition to the inclusion of a larger sample size, this study also expanded the DDR gene panel investigated based on recent studies involving PARP inhibitors [18]. The investigators aim to demonstrate a significant prevalence of DDR gene mutations in the genomic landscape of HNSCC which may assist in laying groundwork for NGS-guided investigations of PARP inhibitors in HNSCC. Correlation of patient characteristics and outcomes of tDNA and ctDNA sequencing results was also performed to assist in identification of patients with HNSCC likely to benefit from NGS.

## 2. Materials and Methods

This study is a single-institution retrospective review of adult patients with HNSCC who underwent NGS (tDNA, ctDNA or both) at Wake Forest Baptist Health between August 2014 and October 2020. The Wake Forest School of Medicine Institutional Review Board approved the study (IRB00057787). HNSCC patients were required to have had a valid tDNA and/or ctDNA test to be included in the study. Patients with cutaneous SCC or salivary gland cancers, as well as patients with other active primary cancers, were excluded.

Eighteen DDR genes (*BRCA1, BRCA2, ATM, BRIP1, BARD1, CDK12, CHEK1, CHEK2, FANCL, PALB2, PPP2R2A, RAD51B, RAD51C, RAD51D, RAD51L, APC, ARID1A* and *MLL3*) were selected for this study based on their involvement with HRR or the FA pathway [16,17,18,19,20]. All 18 genes were tested for tDNA mutations (substitutions, insertion and deletion alterations) by the FoundationOne platform (Foundation Medicine, Cambridge, MA, USA) (FM). Mutations in ctDNA (single nucleotide variants, including indels and fusion alterations) were tested for by the Guardant360 platform (G360) (Guardant Health, Redwood City, CA, USA). Variants of unknown significance were included in this analysis. Six of the eighteen genes selected for this study (*APC, ARID1A, APC, BRCA1, BRCA2* and *CDK12*) are included in the G360 platform and were analyzed for ctDNA mutations.

Concordance analysis was performed for the six genes sequenced by both FoundationOne and Guardant360 platforms. Concordance was calculated per patient at the gene level. Full concordance is defined as detection of matching, identical mutations in tDNA and ctDNA per gene, per patient. Partial concordance is defined as detection of identical mutations in tDNA and ctDNA and additional mutations in tDNA and/or ctDNA within a gene. Discordance is defined as detection of different mutations by tDNA and ctDNA in a gene.

Demographic and disease characteristics were collected from the electronic medical record with regard to age (grouped as older and younger than the median age), gender, stage of disease at diagnosis (per cancer staging AJCC 8th edition), HPV status defined by HPV by PCR and/or p16 status, smoking status (grouped as never-smokers vs. ever-smokers where ever smokers were defined as former or current smokers), alcohol use, tumor subsite (oral cavity, oropharynx, larynx, hypopharynx, nasopharynx, paranasal sinuses or unknown primary) and treatment received before tDNA and before ctDNA collection (chemotherapy, radiotherapy or both). Outcome measures included overall survival measured from the time of diagnosis, from the time of tDNA collection or from the time of ctDNA collection. Survival at 1 and 2 years measured from the date of tDNA or ctDNA collection, survival at the end of the study and extent/burden of disease at last visit were also included in outcome data. It should be noted that, for all calculations in which extent of disease was measured, three categories were considered. These were defined as “no evidence of disease”, “localized disease” and “metastatic disease.” Patients with follow-up shorter than 6 months from the date of last NGS testing were excluded from the outcome analysis.

Subset analysis was performed for the six genes (*ATM, APC, ARID1A, BRCA1, BRCA2* and *CDK12*) for which alterations could be detected in both tDNA and ctDNA via the above methods (6-gene subset). Additional subset analyses were conducted for *BRCA1* and *BRCA2* genes (2-gene subset), for which PARP inhibitors are FDA-approved in patients with mutations present in breast, ovarian and pancreatic cancer, and for *BRCA1, BRCA2* and *ATM* (3-gene subset), for which PARP inhibitors have been recently approved when such mutated genes are identified in prostate cancer. The gene subsets can be reviewed in (Table 1). Patients were considered positive for a DDR gene mutation if they had a mutation in one or more DDR gene mutations in tDNA, ctDNA or tDNA and/or ctDNA.

### Statistical Analysis

Descriptive statistics of means and standard deviations were calculated for continuous variables. Counts and percentages for categorical variables were also presented. There was notation of the prevalence of mutations in each of the eighteen selected genes. Several sets of these results were created based upon the genetic material in which the mutation was detected (tDNA only, ctDNA only and tDNA ± ctDNA). Composite measures were then created to determine whether mutations were present in any of the gene subsets (2-gene, 3-gene or 6-gene). For each of these dichotomous variables, groups of patients with or without mutated DDR were compared with categorical variables using Fisher’s exact tests when both variables were binary. Chi-square tests were used when comparing groups with more than two categories. For analyses comparing mean values of continuous measures, we used two-sample *t* tests. When comparing survival curves, Kaplan–Meier curves were generated and compared groups of patients with DDR mutations to those without using log-rank tests. For some survival models, groups were compared after accounting for a stratification variable, such as staging at diagnosis or HPV status. Cox proportional hazards regression models were used to examine the relationship of survival (from time of sample collection) to a number of potential risk factors and predictors in the same model. Age, tobacco use, tumor site, nodal stage at diagnosis and previous treatment with combined chemoradiation therapy were included in these adjusted models based on statistical significance and/or clinical importance (i.e., age was included despite not having been found to be statistically significant based on clinical relevance). Hazard ratios and corresponding 95% confidence intervals were estimated from these proportional hazards regression models. In all analyses, two-sided tests with an alpha level of 0.05 were used to determine significance. SAS version 9.4 (SAS Institute, Cary, NC, USA) was used to perform all analyses.

## 3. Results

### 3.1. Patient Characteristics

One hundred and seventy total patients met criteria for enrollment. Of these, 139 underwent NGS via tDNA, 146 via ctDNA and 115 via both methods. Demographics and disease characteristics are available for review in Table 2. Age, race, gender and stage in this study are congruent with a standard HNSCC population.

### 3.2. Sequencing Results. Prevalence of Mutations in DDR Genes in Study Population

**Figure 1:**
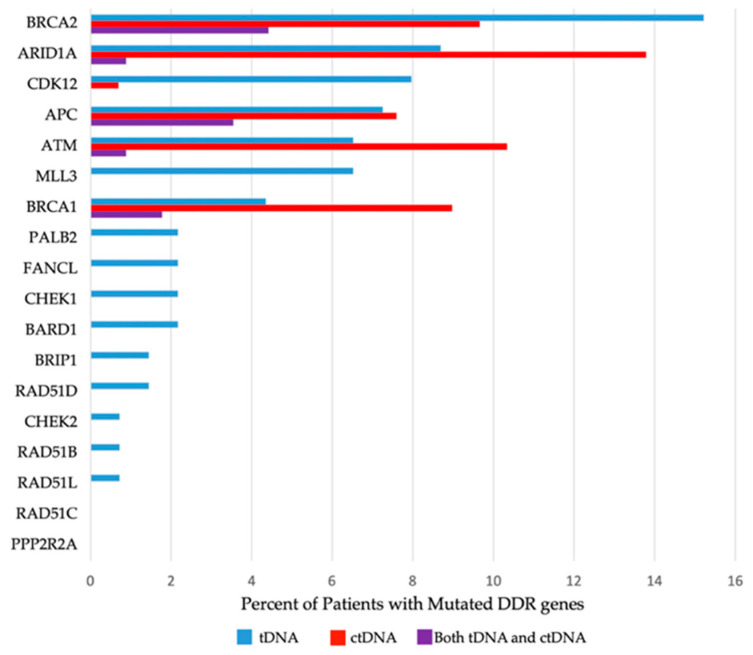
Histogram of Gene Prevalence. ctDNA, circulating tumor DNA; DDR, DNA damage repair; tDNA, tumor tissue DNA.

**Figure 2:**
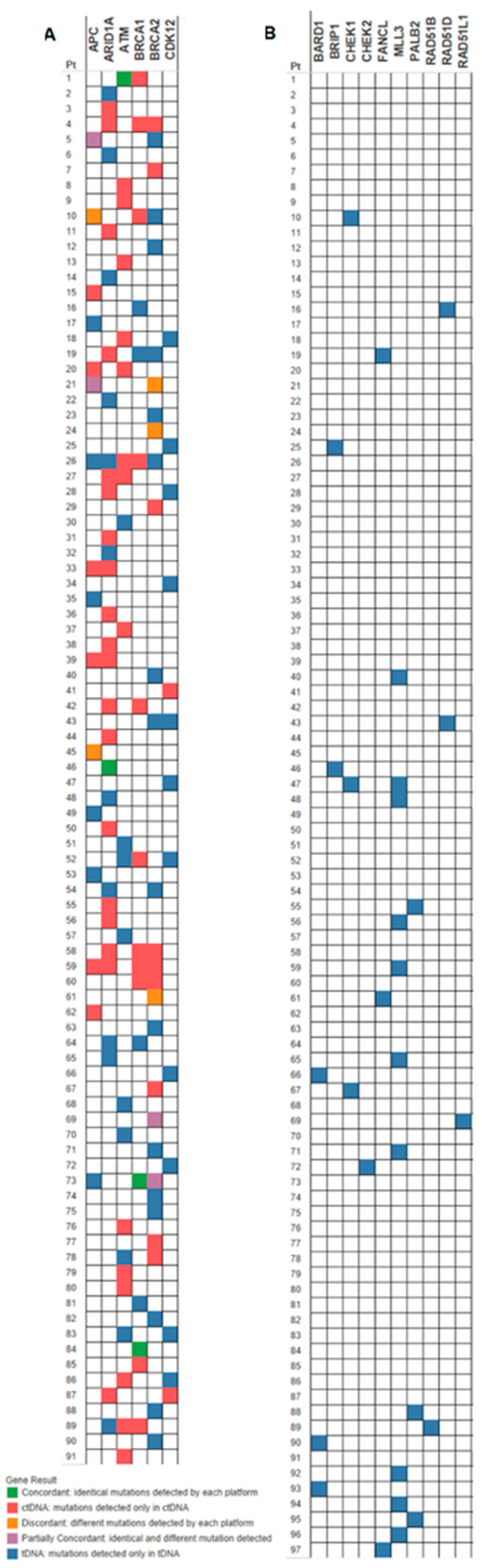
(**A**) Mutated genes tested in both tDNA and ctDNA. (**B**) Mutated genes tested in tDNA only. Green boxes indicate concordant mutations (identical mutations detected by the two platforms). Gold boxes indicate discordant mutations (different mutations reported by each platform in the same gene). Purple boxes represent partially concordant mutations (concordant and discordant mutations reported by the two platforms in the same gene). Red boxes indicate mutations detected in ctDNA only. Blue boxes indicate mutations that were found in tDNA only.

Presence (or absence) of mutated DDR genes was reported per patient, stipulating the specific DDR gene mutated and sample source (ctDNA and/or tDNA). Detailed information about the prevalence of specific mutated DDR genes can be located in Table 3 and in Figure 1, and the allocation of the DDR gene mutations among patients can be viewed in Figure 2.

We found that 97 of the 170 patients (57.1%) had one (or more) mutations in one (or more) DDR gene(s) detected in either ctDNA and/or tDNA samples; 81 patients (47.6%) had mutations in at least one of the genes in the 6-gene subset (*ATM, APC, ARID1A, BRCA1, BRCA2* or *CDK12*). A total of 70 patients (41.1%) had mutations in *BRCA1, BRCA2* and/or *ATM* (the 3-gene subset) detected in ctDNA and/or tDNA (Figure 2), and 47 patients (27.6%) had ctDNA and/or tDNA mutations in *BRCA1* and/or *BRCA2* (2-gene subset) (Figure 1 and Table 3). The most frequently mutated DDR genes in the study HNSCC population were *BRCA2* and *ARID1A*, both mutated in 17.6% of the patients tested by either tDNA and/or ctDNA. *ATM* and *BRCA1* followed, with mutations identified in 13.5% and 10% of patients, respectively.

In total, 139 patients underwent tDNA testing. All genes in the 18-gene panel were included in tDNA testing: 66 of the patients tested (47.5%) had at least one tDNA mutation in the 18-gene panel; 55 patients (39.5%) had tDNA mutations in the 6-gene subset; 34 (24.4%) and 25 (17.9%) patients had tDNA mutations in the 3-gene and 2-gene subsets, respectively (Figure 1 and Table 3).

Out of the 18 DDR genes tested for mutations in tDNA, 16 were found to be mutated in one or more patients. Mutations in *PPP2R2A* and *RAD51C* were not detected in any patients. The most frequently mutated DDR genes, on a per patient basis, in tDNA were *BRCA2* (21 patients), *ARID1A* (12 patients), *APC* (10 patients), *CDK12* (11 patients) and *MLL3* (10 patients), respectively (Table 3). The most frequently altered gene in the tDNA analysis overall was *BRCA2*, with 46 mutations in 21 patients. The gene with the highest number of alterations in a single patient was *MLL3*, with 4.2 mutations detected in tDNA. Remarkably, one patient had 9 mutations in *BRCA2* and 9 mutations in *FANCL* gene in the tDNA analysis (Figure 1 and Table 3).

In total, 146 patients underwent ctDNA testing: 54 of these patients (37.0%) had at least one ctDNA mutation in the total gene panel assessed by the chosen platform; 34 of the patients who underwent ctDNA testing had ctDNA mutations in the 3-gene subset, and 22 of the patients who underwent ctDNA testing had ctDNA mutations in the 2-gene subset (Table 3).

All six DDR genes included in the panel (*BRCA1, BRCA2, ATM, APC, ARID1A* and *CDK12*) were found to be altered in at least one patient. The most frequently mutated DDR genes in ctDNA were *ARID1A* (19 patients), *ATM* (15 patients), *BRCA2* (14 patients) and *BRCA1* (13 patients) (Table 3). The most frequently altered gene in the ctDNA analysis was *ARID1A* with 23 mutations in 19 patients.

For the 115 patients with both tDNA and ctDNA results available, concordance of mutations among the six DDR genes common to both assays, per patient, is depicted in the oncoprint (Figure 2A). About 4.1% of patients had mutations that were concordant, 4.1% had partial concordance and 5.2% were discordant. Close to half (44.3%) of patients who underwent tDNA and ctDNA testing had only tDNA mutations, and 32% of patients had only ctDNA mutations. The mutations in the genes analyzed by FoundationOne only, per patient, are depicted in part B of the oncoprint (Figure 2B).

### 3.3. Pathogenic and Presumed Pathogenic Mutations

Pathogenic or presumed pathogenic mutations, as depicted in FM and G360 reports and described as “deleterious” or “inactivating,” were reported in a total of 30 of the 170 study patients (17.6%) in ctDNA and/or tDNA: 16 of the 139 (11.5%) patients for whom tDNA samples were tested were found to have pathogenic or presumed pathogenic mutations, and 18 of the 146 (12.3%) for whom ctDNA samples were tested were identified as having such mutations in DDR genes. Only 4 of the 30 patients had pathogenic mutations identified in both tDNA and ctDNA, with a significant 40% of the patients being identified exclusively by ctDNA testing (Table 3).

### 3.4. Targetable Mutations

FM and G360 reported availability of off-label clinical protocols with PARP inhibitors for pathogenic mutations in *BRCA1, BRCA2, ATM* and, more recently, in *PALB2, ARID1A* and *CDK12*. Therefore, pathogenic or presumed pathogenic mutations in these genes were deemed “targetable” with PARP inhibitors in this study. Based on the information provided by FM and G360, 27 patients (15.9%) of the study patients would be eligible for off-label therapy with a PARP inhibitor, with 13 patients (9.3% of the tDNA tested patients) and 17 patients (11.6% of the ctDNA tested patients) being potential candidates (Table 4). *ATM* was the DDR gene with the highest number of pathogenic mutations reported in 11 patients (6.4% of the 170 patients tested by ctDNA and/or tDNA); 9 of the 11 patients were identified by ctDNA testing. *BRCA1* and *ARID1A* followed, with 6 and 5 patients, respectively, identified with targetable mutations, with the majority of patients identified again by ctDNA testing (5 and 3 patients, respectively) (Table 4).

### 3.5. Prevalence of DDR Gene Mutations across Demographic Groups

Patients were deemed as either positive or negative for mutated DDR genes in the 18-gene panel (all genes) or for ctDNA, tDNA or either (ctDNA and/or tDNA) in each of the subsets. No significant association was found between patients with mutated DDR genes in the 18-gene panel and age, gender, race, smoking status, alcohol use or stage at diagnosis. Patients with mutated DDR genes within the 3-gene subset in ctDNA and in either/both tDNA and/or ctDNA were statistically more likely to be older than the median patient age of 60 years (*p* values of 0.04 and 0.050, respectively). No other associations with age, gender, race, smoking status, alcohol use or stage at diagnosis were found in any of the other subsets.

Patients with DDR gene mutations detected in ctDNA and/or tDNA were associated with HNSCC subsite (*p* = 0.02) in the 18-gene panel analysis. Laryngeal primaries, specifically, had a higher presence of DDR gene mutations detected in this gene set detected in ctDNA (*p* = 0.02), tDNA (*p* = 0.06) or via in ctDNA and/or tDNA method (*p* = 0.01). Oropharyngeal primaries correlated with a lower prevalence of DDR gene mutations in patients detected in tDNA (*p* = 0.06) and in tDNA and/or ctDNA (*p* = 0.01). Statistical significance of the lower prevalence of patients with DDR gene mutations in oropharyngeal cancers was preserved in the 6-gene subset analysis (*p* = 0.04 for tDNA, and *p* = 0.01 for tDNA and/or ctDNA), in the 3-gene subset analysis (*p* = 0.01 for tDNA; *p* = 0.054 for ctDNA; and *p* < 0.01 for tDNA and/or ctDNA) and in the 2-gene subset analysis (*p* = 0.02 for tDNA, and *p* = 0.03 for tDNA and/or ctDNA). The 3-gene subset analysis showed an association in which patients with DDR gene mutations detected via tDNA and/or ctDNA were more likely to have more advanced disease at time of diagnosis with respect to advanced cancer stage (I–IV) (*p* = 0.06), N stage (N0 to N3) (*p* = 0.02) and within stage IV disease (between groups A, B and C) (*p* = 0.03). N stage also correlated significantly with the prevalence of patients with ctDNA mutations (*p* = 0.02).

Patients treated with chemotherapy, radiotherapy or both before collection of ctDNA had a significantly higher presence of DDR gene mutations in ctDNA (*p* < 0.01). Data also indicated an increased prevalence of mutations in tDNA and/or ctDNA in patients treated before tDNA collection (*p* = 0.03).

### 3.6. HPV and Smoking Status and the Prevalence of DDR Genes Mutations

HPV and/or p16 testing was available for 123 (72.35%) patients. HPV and/or p16 were negative in 65 patients (52.84% of those tested) and positive in 58 patients (47.15% of those tested). Positive HPV and/or p16 tumors were associated with increased probability to be alive at the end of the study (*p* < 0.01) and with tendency for better OS measured from the time of diagnosis (*p* = 0.06). No significant correlation between HPV status and presence of a DDR gene mutation on a per patient basis were discovered in the 18-gene analysis. In the 6-gene subset, however, patients without mutations in tDNA and/or ctDNA were found to be more likely to have HPV-positive disease (*p* = 0.04).

Information about smoking status was available for all patients included in the study: 48 of the 170 patients (28.2%) were never-smokers, and 122 patients (71.8%) were ever-smokers. A nearly significant lower presence of ctDNA DDR gene mutations was found in non-smokers compared with ever-smokers (*p* = 0.06) in the 3-gene subset analysis. Non-smokers had a nearly significant better chance to be alive at the end of the study (*p* = 0.058) and a significantly better OS measured from the time of diagnosis (*p* = 0.03) when compared to ever-smokers.

### 3.7. Survival Analysis

**Figure 3:**
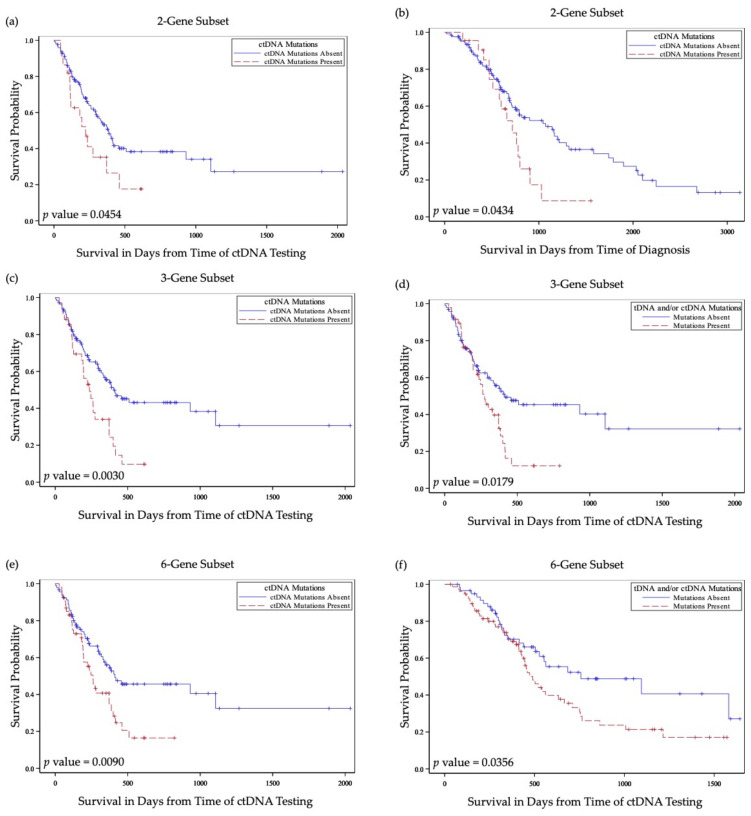
Kaplan–Meier curves depicting survival differences in patients with DDR gene mutations in comparison to those without DDR gene mutations. (**a**) Survival from time of ctDNA testing in patients with vs. without DDR genes mutations in ctDNA in the 2-gene subset. (**b**) Survival from time of diagnosis in patients with vs. without DDR genes mutations in ctDNA in the 2-gene subset. (**c**) Survival from time of ctDNA testing in patients with vs. without DDR genes mutations in ctDNA in the 3-gene subset. (**d**) Survival from time of ctDNA testing in patients with vs. without DDR genes mutations in tDNA and/or ctDNA in the 3-gene subset. (**e**) Survival from time of ctDNA testing in patients with vs. without DDR genes mutations in ctDNA in the 6-gene subset. (**f**) Survival from time of ctDNA testing in patients with vs. without DDR genes mutations in tDNA and/or ctDNA in the 6-gene subset. ctDNA, circulating tumor DNA; tDNA, tumor tissue DNA; blue solid lines indicate survival curves for patients without mutations in any of the selected gene panel; red dashed lines indicate survival curves for patients with at least one mutation in any of the selected gene panel.

All patients had at least 6 months of follow up after the last sample collection for NGS. Median follow-up time was 615.5 days from the time of diagnosis and 232.5 days from the time of ctDNA testing. Median survival from the time of diagnosis was 820 days (95% CI 752 to 1140 days) and 372 days (95% CI 262 to 416 days) from the time of ctDNA testing. At last visit, 35.8% of patients had no evidence of disease, 28.4% had recurrent or progressive loco–regional disease, and 35.8% had metastatic disease (Table 2). Overall, patients with mutations in DDR genes had poorer prognosis (Table 5 and Figure 3).

### 3.8. Prognostic Value of Presence of ctDNA Mutations in DDR Genes

Patients without ctDNA DDR gene mutations in the 6-gene subset or in the 3-gene subset were significantly more likely to be alive at the end of the study (*p* = 0.04, and *p* = 0.01, respectively). Similarly, patients without ctDNA mutations specifically in BRCA2 or in APC genes were more likely to be alive at the end of the study (*p* = 0.01, and *p* = 0.01, respectively). Patients with ctDNA mutations in DDR genes in the 6-gene and in the 3-gene subsets were more likely to have a more advanced cancer status at the last visit (*p* = 0.03, and *p* = 0.01, respectively). Presence of mutated DDR genes in ctDNA was also associated with significantly worse 2-year survival (*p* < 0.01).

Patients with ctDNA DDR gene mutations had significantly worse overall survival measured from the time of ctDNA collection (*p* = 0.01) (Figure 3a). This relationship remained statistically significant in a Cox proportional hazards regression model when adjusted for age, tobacco use, tumor site, nodal stage at diagnosis and previous treatment with combined chemoradiation therapy in a multivariate analysis model (*p* = 0.053) (Table 6). Similar associations with overall survival were found in studies for patients with ctDNA DDR gene mutations in 3-gene and 2-gene subsets in the univariate (*p* < 0.01, and *p* = 0.04, respectively) and in the multivariate (*p* = 0.02, and *p* = 0.04, respectively) analyses (Table 5 and Table 6 and Figure 3c,e). Association with overall survival measured from the time of diagnosis was statistically significant for patients with ctDNA mutations in the 3-gene and 2-gene analysis (*p* < 0.01, and *p* = 0.04, respectively (Table 5 and Figure 3f).

### 3.9. Prognostic Value of Presence of tDNA Mutations in DDR Genes

A patients’ possession of tDNA DDR gene mutations showed no significant prognostic value when analyzed for correlation with disease status at the end of the study, survival at 1 or 2 year(s) or with overall survival. tDNA DDR gene mutations present specifically in APC or in CDK12 genes were associated with decreased likelihood to be alive at the end of the study (*p* = 0.01, and *p* = 0.01, respectively).

### 3.10. Prognostic Value of Presence of tDNA and/or ctDNA Mutations in DDR Genes

Patients with mutations in one or more DDR genes in the 6-gene and 3-gene subsets detected in tDNA and/or ctDNA were significantly more likely to have a greater extent of disease at last visit (*p* < 0.01, and *p* = 0.01, respectively) (Table 5). In the 6-gene subset analysis, patients with mutated DDR genes had significantly decreased overall survival measured from the time of ctDNA collection (*p* = 0.053) or from the time of tDNA collection (*p* = 0.04) (Figure 3b) and did not reach significance when measured from the time of diagnosis (*p* = 0.07) (Table 5). For the 3-gene subset analysis, patients with mutations in tDNA and/or ctDNA were also found to have decreased overall survival when measured from the time of ctDNA collection (*p* = 0.02) (Figure 3d) and did not reach significance when measured from the time of tDNA collection (*p* = 0.07).

Mutations present in APC or CDK12 genes, individually, were again associated with decreased likelihood to be alive at the end of the study (*p* = 0.01, and *p* = 0.01, respectively) when measured in tDNA and/or ctDNA. Interestingly, mutations in ARID1A as well as in MLL3 were associated with improved chance to be alive at the end of the study when measured in tDNA (*p* = 0.04, and *p* = 0.06, respectively) or in both tDNA and/or ctDNA for MLL3 gene (*p* = 0.053).

## 4. Discussion

This study is a single-institution retrospective analysis examining the prevalence, prognostic and potential therapeutic implications of DDR gene mutations in tDNA and ctDNA in a dedicated cohort of HNSCC patients. To the authors’ knowledge, this study is the first to elucidate the significance and the role of the genomic profile of DDR genes in the HNSCC when evaluated by both tDNA and ctDNA analysis, alone, or in combination.

The selection of the 18 genes chosen for this study was based on literature review of genes’ roles and importance in DDR pathways as well as on inclusion as biomarkers in clinical studies [17]. A subset was created for further analysis based on the testing profile available for both tDNA and ctDNA (6-gene subset). Creation of other subsets was based on potential clinical therapeutic utility, with 2-gene subset and 3-gene subset reflecting the gene biomarkers utilized for approval of PARP inhibitors in the management of breast, ovarian, pancreatic and prostate cancers. Variants of unknown significance were not excluded from data related to prevalence, analysis of demographics or prognostic associations. The decision to include these mutations was based on the notion that there was insufficient scientific data to dismiss them and that their clinical significance may become apparent in the future. However, variants of unknown significance were excluded from the reporting of the genes identified as potential targetable mutations in current clinical protocols.

The population studied in this analysis is consistent with a standard HNSCC population in terms of age, gender, race, smoking status and prevalence of HPV driven disease. A previous study on a fraction of patients in this group (75 of the 170 patients) has demonstrated a prevalence of mutations in *TP53, CDKN2A, TERT, BRCA2* and *NOTCH1* similar to other reported populations [3,15,17,24,25]. Conventional prognostication tools, including those related to HPV or smoking-driven diseases held true in this analysis. Non-smokers and those with HPV/p16 positivity had a significantly better overall survival and were more likely to be alive at the end of the study.

Data presented in this analysis demonstrates a higher than previously reported prevalence of DDR gene mutations in HNSCC. In this analysis, 47.4% had at least one tDNA mutation and 37% had at least one ctDNA mutation in the selected gene profile. *BRCA2* and *ARID1A* were the two DDR genes with the highest prevalence in our HNSCC population: both mutated in 17.6% of the patients tested in either tDNA and/or ctDNA. *ATM* and *BRCA1* were the next most common and were found to be mutated in 13.5% and 10% of patients, respectively. Other studies have reported a lower frequency of such mutations. For example, one such study reported 6% for *BRCA1* and 7% for *BRCA2*. It should be noted that such studies utilized only tumor tissue for NGS evaluation [6,26,27,28,29].

When variants of unknown significance and mutations thought to not influence gene function were excluded, pathogenic or presumed pathogenic mutations in DDR genes were reported in 29 of the 170 study patients (17%). A total of 11.5% of tDNA samples and 12.3% of ctDNA samples were found to have such mutations. These results compare well with the DDR gene mutation profile reported by other studies. For example, Heeke et al. studied genes involved in homologous recombination across multiple tumor types with the most frequently mutated genes overlapping with our study (*ARID1A*, *BRCA2, BRCA1*). Overall, pathogenic mutations in genes involved in homologous recombination were found in 17.6% of the 17,566 tumors tested and 6.8% of a total of 206 head and neck tumors [26]. With a variation in the selection of the less frequently mutated genes and with the addition of ctDNA testing, this study has significantly increased the percentage of theoretically actionable mutations in the HNSCC, to 17%. Addition of ctDNA to this report increased the yield of NGS by nearly two-fold when compared to tDNA testing, alone. Concordance results also supported the use of both NGS analysis methods in combination. Concordance was limited in the DDR genes analyzed, and, in more than 90% of the patients, each method brought complimentary information, increasing the yield to identify patients for precision oncology treatments. It is noteworthy that the DDR gene with the highest incidence of targetable mutations in this study is *ATM*, with pathogenic mutations reported in 6.4% of the 170 patients tested for NGS and with 9 of the 11 patients being identified by ctDNA testing. Next, *BRCA1* was identified with targetable mutations in 6 patients, with majority of patients identified again by ctDNA testing, and *ARID1A* in 5 patients.

This analysis demonstrated that several groups were predisposed to DDR gene mutations. For example, patients older than the median (60 years) were more likely to have mutations in *ATM, BRCA1* and/or *BRCA2* (the 3-gene subset) detected in ctDNA or in ctDNA and/or tDNA. Certain HNSCC subsites were more likely to have mutations in DDR genes (laryngeal primaries) and others less likely (oropharyngeal) when tested in tDNA or ctDNA or both. Analysis of the gene subsets also showed decreased prevalence of DDR gene mutations in oropharyngeal cancer. Patients with more advanced disease stage (stages I to IV), and those with more advanced stage IV disease (between groups A, B and C) were more likely to have mutations in *ATM, BRCA1* and/or *BRCA2* (the 3-gene subset) detected via tDNA and/or ctDNA. N stage (N0 to N3) also correlated significantly with the prevalence of ctDNA mutations in the 3-gene subset analysis. Patients treated with chemotherapy, radiotherapy or both prior to collection of their genetic samples were more likely to have DDR gene mutations in ctDNA or in samples collected by either method. To the authors’ knowledge, it is the first time that these demographic correlations were identified in the study of DDR genomic profile in HNSCC, and comparative studies are not available for validation.

The 6-gene subset analysis in this study demonstrated a significantly lower prevalence of mutations in tDNA and/or ctDNA DDR genes in HPV-positive disease. All other subset analyses in tDNA and/or ctDNA support these findings, without reaching statistical significance. These results are further supported by other data in this report. Non-smokers vs. ever-smokers were also less likely to have gene mutations in the 3-gene subset analysis. In addition, given that fewer mutated DDR genes (such as in HPV-positive patients and in non-smokers) were found to be associated with improved survival, it is congruent with the HPV mutation results. This is in agreement with studies that have demonstrated increased expression (i.e., increased presence of functional copies) of DNA repair genes in HPV-positive HNSCC [30]. Two other studies reporting results from genomic cohorts originating from the University of Chicago and University of Michigan (120 and 34 patients, respectively) described that mutations in DDR genes and Fanconi Anemia genes (a spectrum that contains important overlapping genes), respectively, were more frequently associated with HPV positivity [27,31]. Differences in definition of HPV phenotype, in NGS techniques and in DDR gene panel selection could account for the discordant results. Additional effects of confounding variables, such as smoking status, age, stage of disease and previous treatment(s) could further complicate the relationship between HPV status and gene mutations.

Presence of mutated DDR genes was found to be a compelling indicator of poor prognosis. Strong statistically significant correlations were noted between the presence of DDR gene mutations and decreased overall survival when measured from the time of genetic sample collection or from time of diagnosis in ctDNA (in all subsets) and in tDNA and/or ctDNA in selected subsets (Table 3). The relationship between ctDNA DDR mutations and overall survival remained statistically significant in a Cox proportional hazards regression model when adjusted for age, tobacco use, tumor site, nodal stage at diagnosis and previous treatment with combined chemoradiation therapy in all subsets. No similar correlation was found between tDNA mutations in DDR genes and prognosis. Existing literature suggests that expression of certain DDR genes, including *BRCA1* and *BRCA2*, is associated with increased survival in HNSCC patients as the preservation of efficient repair mechanisms maintains genomic stability [32]. Similarly, another study has listed *BRCA1* expression, alone, to be indicative of survival in HNSCC [30]. As another indicator of poor prognosis, patients with DDR gene mutations were significantly more likely to have more advanced disease burden at the time of the last visit, as measured in ctDNA and in both tDNA and/or ctDNA in the 6-gene and 3-gene subsets.

Overall, statistically significant associations between the presence of mutated DDR genes and demographic variables and/or survival were more frequently identified in ctDNA rather than in tDNA. This possibly reflects differences in sampling and in NGS techniques. Challenges in tissue sample acquisition and appraisal, including availability and tumor heterogeneity, are universal to tDNA studies. Likewise, studies regarding ctDNA have uncovered that liquid biopsies do not reflect the complete mutation profile of the tumor, either, and such studies have noted increased sensitivity with increased burden of disease [15,33]. It is also feasible that differences in sequencing results between samples are also reflective of the different time points at which the samples were collected (ctDNA studies were typically performed after tDNA studies in this cohort) and, therefore, may be impacted by tumor progression, interim treatments, etc. Differences between the FoundationOne and Guardant360 sequencing techniques may affect the concordance of DDR gene mutation results and, therefore, the correlation with different clinical variables.

The high prevalence of DDR gene mutations in this cohort detected in ctDNA samples, tDNA samples or both is of considerable clinical interest, as mutations in these genes are potential targets for novel cancer treatments, including PARP inhibitors. FoundationOne and Guardant360 report off-label clinical protocols with PARP inhibitors for pathogenic or presumed pathogenic mutations in *BRCA1, BRCA2, ATM* and, more recently, in *PALB2, ARID1A* and *CDK12*. No off-label clinical protocols with PARP inhibitors were reported for mutations in *APC* or *MLL3*; therefore, patients with such mutations were not included here. A total of 15.9% of the 170 study patients would be eligible for off-label PARP1 inhibitor clinical protocols, with 9.3% of tDNA-tested patients and 11.6 % of ctDNA-tested patients being potential candidates. These frequencies rival reported frequency of these mutations in breast (15.6%), ovarian (20.0%), prostate (14.1%) and pancreatic cancers (15.4%) for which PARP inhibitors are currently FDA approved therapeutics [26]. 

Notably, this report emphasizes the utility of ctDNA testing by demonstrating improved sensitivity in the identification of patients who might benefit from targeted drug therapy. Only 3 of the 27 patients identified with presumed targetable mutations for PARP inhibitors were identified in both tDNA and ctDNA, with more than half (14) of the patients being identified exclusively by ctDNA testing. These results support efforts made in the field of precision oncology to revolutionize the treatment of HNSCC, with consideration for targeted, mutation-guided clinical protocols with single agent PARP inhibitors. Review of the literature revealed only one study that evaluated efficacy of a single agent PARP inhibitor, Olaparib, in a limited number of pre-operative HNSCC cases. In this study, Olaparib was used with or without cisplatin or durvalumab. The report concluded that mutations in DDR genes were associated with sensitivity to Olaparib in HNSCC, as has previously been demonstrated in other malignancies [34]. Additional ongoing clinical trials for treatment of HNSCC with PARP inhibitors rely on combination therapy in which chemotherapy and/or radiotherapy are used to sensitize tumors to PARP inhibitors. Such studies find basis in pre-clinical trials in which synergy was noted between PARP inhibitors and more conventional therapies [35]. These studies and others highlight the tolerability and effectivity of PARP inhibitors in HNSCC but are all in small cohorts, and none uses NGS to guide therapy [21,22,23]. The strong correlation of the presence of DDR gene mutations with poor survival in this study raises the possibility that NGS-guided treatment with PARP inhibitors in HNSCC might lead to improvement in survival in select patients. 

This is among the largest cohorts of patients with HNSCC in whom tDNA mutations were studied and is the only report in which DDR gene mutations were analyzed in a relatively large HNSCC population by ctDNA, alone, or in combination with tDNA. Findings from this report support further use of ctDNA analysis to predict prognosis and to increase sensitivity in the detection of targetable mutations and underscore further investigations into PARP inhibitors for the treatment of HNSCC. This report has a number of limitations. Data was collected from a single institution and geographic area. Furthermore, dependence on the electronic medical record, self-reported data (for smoking and alcohol use) and utilization of commercially available NGS platforms with differences in technical approaches introduced error that could not be corrected. Finally, this correlative data does not imply causation, therefore limiting the number and types of conclusions that can be drawn.

### Future Directions

This study notably demonstrates both the high prevalence of DDR gene mutations in HNSCC and the poor prognosis associated with such mutations. The increased prevalence of DDR gene mutations measured in this study was the result of combining tDNA with ctDNA testing. The low overall concordance between tDNA and ctDNA samples, and the significant contribution of ctDNA testing to the number of identified mutations targetable with PARP inhibitors, supports using the combination of the two methods in future clinical practice to raise the sensitivity of genetic testing. These results are expected to urge the advancement of clinical research with NGS-guided use of PARP inhibitors in the treatment of HNSCC, rather than the non-targeted combination with other treatment modalities, which is currently the only approach to PARP inhibitors utilization in the management of HNSCC. The indisputable association of ctDNA mutations in DDR genes with poor prognosis and survival in HNSCC further supports the acceleration of investigating PARP inhibitors in the management of HNSCC with the future goal to improve survival in this group of patients with notable poor prognosis. Expansion of the DDR gene panel to be tested for mutations should be considered in the future.

## 5. Conclusions

Despite the benefits of NGS in the management of many malignancies, the mutational landscape of HNSCC remains largely undescribed. This study is the largest cohort to date to analyze the genomic landscape in both blood and tumor tissue in patients with HNSCC and reports a high prevalence of DDR gene mutations in this tumor type. Utilizing both ctDNA and tDNA analysis, the incidence of targetable mutations in this HNSCC cohort was found comparable with other cancers such as breast, ovarian, prostate and pancreatic cancers for which PARP inhibitors are now standard of care. For the first time, the addition of ctDNA analysis contributed to the identification of an increased incidence of DDR gene mutations in patients older than 60 years, in laryngeal primaries, in patients with advanced stage at diagnosis and in patients with tumors previously treated with chemotherapy and/or radiotherapy, while the incidence was found significantly decreased in oropharyngeal cancer and in HPV-positive patients. Patients with DDR gene mutations in ctDNA rather than tDNA had significantly worse prognoses, with more advanced disease burden at the end of the study and with decreased overall survival in univariate analysis and in Cox proportional hazard regression models adjusted for statistically and/or clinically significant variables. These results are expected to prompt further clinical investigations with NGS-guided PARP inhibitors for the treatment of HNSCC.

## Figures and Tables

**Table 1:** 18-Gene panel and gene subsets.

DNA Damage Repair Genes			
18-Gene Panel (Selected based on literature review)	6-Gene Subset (Genes common to both tDNA and ctDNA assays)	3-Gene Subset (Mutated genes with approved PARP inhibitors in prostate cancer)	2-Gene Subset (Mutated genes with approved PARP inhibitors in ovarian, breast and pancreatic cancer)
*BRCA1, BRCA2, ATM, BRIP1, BARD1, CDK12, CHEK1, CHEK2, FANCL, PALB2, PPP2R2A, RAD51B, RAD51C, RAD51D, RAD51L, APC, ARID1A, MLL3*	*ATM, APC, ARID1A, BRCA1, BRCA2, CDK12*	*BRCA1, BRCA2, ATM*	*BRCA1, BRCA2*

ctDNA, circulating tumor DNA; tDNA, tumor tissue DNA.

**Table 2:** Patient Characteristics.

Characteristics	No.	%	Characteristics	No.	%
Age at Diagnosis (Years)			Primary Tumor Location		
Median	60	-	Nasopharynx	14	8.2%
≥60	85	50.0%	Oropharynx	68	40.0%
<60	85	50.0%	Oral Cavity	40	23.5%
			Hypopharynx	10	5.9%
Gender			Larynx	27	15.9%
Male	123	72.4%	Sino–Nasal	6	3.5%
Female	47	27.6%	Unknown	5	3.0%
Race			Disease Stage at Time of Diagnosis		
Caucasian	142	83.5%			
African American	19	11.2%			
Other	9	5.3%	Cancer Stage
			I	21	12.4%
ETOH Status			II	30	17.6%
Never	92	54.1%	III	37	21.8%
Former	36	21.2%	IV	82	48.2%
Active	72	24.7%			
			Cancer Stage IV		
Smoking Status			IVA	50	29.4%
Never	48	28.2%	IVB	22	12.9%
Former	50	29.4%	IVC	9	5.3%
Active	72	42.4%			
			N Stage		
HPV and/or p16			N0	47	27.6%
Negative	61	35.9%	N1	35	20.6%
Positive	61	35.9%	N2	69	40.6%
Not Tested	48	28.2%	N3	19	11.2%
tDNA Tissue			Disease Status		
Source			At Last Visit		
Primary Tumor	92	54.1%	No Evidence of Disease	61	35.9%
Regional Lymph Node	11	6.5%	Locoregional	44	25.9%
Metastatic Lesion	11	6.5%	Metastatic (only)	17	10.0%
Recurrence	25	14.7%	Locoregional and Metastatic	48	28.2%

ctDNA, circulating tumor DNA; tDNA, tumor tissue DNA.

**Table 3:** Overall Prevalence of Individual DNA Damage Repair (DDR) Gene Mutations (Any Type of Mutation and Pathogenic or Presumed Pathogenic Mutations).

	Patients with DDR Gene Mutations in tDNA Number (%)		Patients with DDR Gene Mutations in ctDNA Number (%)		Patients with DDR Gene Mutations in tDNA and ctDNA Number (%)		Patients with DDR Gene Mutations in tDNA and/or ctDNA Number (%)	
	Pathogenic Mutation(s)	Any Mutation(s)	Pathogenic Mutation(s)	Any Mutation(s)	Pathogenic Mutation(s)	Any Mutation(s)	Pathogenic Mutation(s)	Any Mutation(s)
All DDR Genes	16 (11.5%)	66 (47.5%)	18 (12.3%)	54 (36.9%)	4 (3.4%)	11 (9.6%)	30 (17.6%)	97 (57.1%)
*BRCA1*	3 (2.2%)	6 (4.3%)	5 (3.4%)	13 (8.9%)	2 (1.7%)	2 (1.7%)	6 (%)	17 (10.0%)
*BRCA2*	3 (2.2%)	21 (15.1%)	1 (0.7%)	14 (9.6%)	0	5 (4.3%)	4 (%)	30 (17.6%)
*ATM*	2 (1.4%)	9(6.4%)	9 (6.2%)	15 (10.3%)	0	1 (0.9%)	11 (%)	23 (13.5%)
*CDK12*	1 (0.7%)	11 (7.9%)	1 (0.7%)	2 (1.4%)	0	0	2 (%)	13 (7.6%)
*APC*	3 (2.2%)	10 (7.2%)	1 (0.7%)	9 (6.2%)	1 (0.9%)	4 (3.4%)	3 (%)	15 (8.8%)
*ARID1A*	3 (2.2%)	12 (8.6%)	3 (2.1%)	19 (13.0%)	1 (0.9%)	1 (0.9%)	5 (%)	30 (17.6%)
*MLL3*	1 (0.7%)	10 (7.2%)	-	-	-	-	-	-
*BRIP1*	0	2 (1.4%)	-	-	-	-	-	-
*BARD1*	0	3 (2.2%)	-	-	-	-	-	-
*CHEK1*	0	3 (2.2%)	-	-	-	-	-	-
*CHEK2*	0	1 (0.7%)	-	-	-	-	-	-
*FANCL*	0	3 (2.2%)	-	-	-	-	-	-
*PALB2*	1 (0.7%)	3 (2.2%)	-	-	-	-	-	-
*PPP2R2A*	0	0	-	-	-	-	-	-
*RAD51B*	0	1 (0.7%)	-	-	-	-	-	-
*RAD51C*	0	0	-	-	-	-	-	-
*RAD51D*	0	2 (1.4%)	-	-	-	-	-	-
*RAD51L*	0	1 (0.7%)	-	-	-	-	-	-
*MLL3*	1 (0.7%)	10 (7.2%)	-	-	-	-	-	-
Total Number of Patients Tested	139 patients		146 patients		115 patients		170 patients	

Occurrences are listed as patients with one or more mutations in the specified gene, rather than total number of mutations encountered for each gene. tDNA was analyzed by the FM platform, which assesses for mutations in all 18 genes. ctDNA was analyzed by the G360 platform, which is limited to analysis of *ATM, APC, ARID1A, BRCA1, BRCA2* and *CDK12*. Pathogenic/presumed pathogenic mutations are as defined by FoundationOne and Guardant 360 reports. The number of patients with mutations in any of the DDR genes does not represent the sum of patients with each individual DDR gene mutations, because a patient could have more than one DDR gene mutated. ctDNA, circulating tumor DNA; DDR, DNA damage repair; tDNA, tumor tissue DNA.

**Table 4:** Prevalence of Clinically Significant and Targetable Mutations.

	tDNA	ctDNA	Both	Off-Label Clinical Protocol with PARP Inhibitors
*BRCA1*	3	5	2	FM, G360
*BRCA2*	3	1	0	FM, G360
*ATM*	2	8 ^1^	0	FM, G360
*ARID1A*	3	2 ^1^	1	G360
*CDK12*	1	1	0	G360
*APC*	2 ^2^	1	1	None
*PALB2*	1	-	-	FM
*MLL3*	1	-	-	None

Occurrences are listed as number of patients with one or more mutations in a gene, rather than total number of mutations encountered for each gene. ^1^ One additional pathogenic mutation was reported in *BRCA1* ctDNA in the same patient who is listed under *BRCA1*. ^2^ One additional pathogenic mutation was reported in *BRCA2* tDNA in the same patient who is listed under *BRCA2*. ctDNA, circulating tumor DNA; FM, FoundationOne Medicine; G360, Guardant 360; tDNA, tumor tissue DNA.

**Table 5:** Correlation of Mutated DDR Genes with Survival Outcomes.

Survival Start Time Point	Overall Survival Univariate Analysis			Overall Survival Adjusted Analysis			1-Year OS	2-Year OS	Survival Last Visit
	HR	95% CI	*p* Value	HR	95% CI	*p* Value	*p* Values		
18-Gene Subset									
tDNA				-	-	-	0.14	0.20	0.71
tDNA collection	0.91	0.57–1.45	0.68						
Diagnosis	0.81	0.51–1.30	0.38						
tDNA and/or ctDNA							0.67	0.75	0.52
tDNA collection	1.20	0.74–1.94	0.46						
ctDNA collection	1.38	0.83–2.29	0.41						
Diagnosis	0.94	0.62–1.44	0.78						
6-Gene Subset									
tDNA				1.62	0.99–2.65	*0.053*	0.46	0.85	0.25
tDNA collection	1.24	0.77–1.97	0.38						
Diagnosis	1.24	0.78–1.98	0.36						
ctDNA							0.10	***<0.01***	***0.04***
ctDNA collection	1.81	1.15–2.85	***0.01***						
Diagnosis	1.38	0.88–2.15	0.16						
tDNA and/or ctDNA							0.40	0.21	***0.01***
tDNA collection	1.68	1.03–2.73	***0.04***						
ctDNA collection	1.56	0.99–2.46	*0.053*						
Diagnosis	1.29	0.85–1.96	0.23						
3-Gene Subset									
tDNA				1.85	1.10–3.12	***0.02***	0.42	0.77	0.92
tDNA collection	1.09	0.63–1.86	0.77						
Diagnosis	1.08	0.63–1.84	0.78						
ctDNA							*0.07*	***0.01***	***0.01***
ctDNA collection	2.04	1.26–3.31	***<0.01***						
Diagnosis	1.99	1.23–3.22	***0.01***						
tDNA and/or ctDNA							0.87	0.16	0.10
tDNA collection	1.55	0.96–2.49	*0.07*						
ctDNA collection	1.73	1.09–2.74	***0.02***						
Diagnosis	1.43	0.94–2.19	0.10						
2-Gene Subset									
tDNA				1.82	0.99–3.38	*0.06*	0.82	0.93	0.64
tDNA collection	0.99	0.53–1.85	0.97						
Diagnosis	0.94	0.50–1.74	0.83						
ctDNA							0.15	***0.04***	0.15
ctDNA collection	1.77	1.00–3.12	***0.04***						
Diagnosis	1.80	1.01–3.21	***0.04***						
tDNA and/or ctDNA							0.89	0.36	0.68
tDNA collection	1.37	0.82–2.29	0.22						
ctDNA collection	1.38	0.83–2.29	0.21						
Diagnosis	1.25	0.77–2.03	0.36						

Results with *p* < 0.05 are bolded in italics; results with 0.05 < *p* < 0.10 are italicized and underlined. Abbreviations: CI, Confidence interval; ctDNA, circulating tumor DNA; HR, hazard ratio; tDNA, tumor tissue DNA.

**Table 6:** Results from Adjusted Cox Proportional Hazard Regression Models of Impact of the Presence of ctDNA Mutations on Overall Survival.

	6-Gene Analysis			3-Gene Analysis			2-Gene Analysis		
	HR	95% CI	*p* Value	HR	95% CI	*p* Value	HR	95% CI	*p* Value
ctDNA mutations	1.62	(0.99–2.65)	*0.053*	1.85	(1.10–3.12)	*0.020*	1.87	(1.02–3.43)	*0.042*
	*p* value for Adjusted Variables			*p* value for Adjusted Variables			*p* value for Adjusted Variables		
Age Below 60 Years Old *(yes* vs. *no)*	0.65			0.70			0.64		
Smoking *(never* vs. *ever)*	0.41			0.54			0.55		
N Stage *(N0 vs N1, N2, N3)*	0.33			0.49			0.31		
Subsite *(OP* vs. *OC*, *Pharynx*, *Other)*	***0.02***			***0.02***			***0.04***		
CRT Prior to ctDNA Test *(yes* vs. *no)*	***<0.01***			***<0.01***			***<0.01***		

Analyses with *p* < 0.05 are bolded in italics and underlined; results with 0.05 < *p* < 0.10 are italicized and underlined. Abbreviations: CI, Confidence interval; ctDNA, circulating tumor DNA; HR, hazard ratio; CI, hazard ratio confidence interval; CS, chi-squared analysis; N/A, non-applicable; CRT, combined chemotherapy and radiation therapy; OC, oral cavity; OP, oropharynx.

## Data Availability

The datasets analyzed are available from the corresponding author upon request.
